# The Breast to Bone (B2B) Cohort Study to Prevent, Detect and Improve Treatment of Metastatic Disease: Baseline Assessment, Description and Progress

**DOI:** 10.3390/ijerph22020242

**Published:** 2025-02-08

**Authors:** Nigel T. Brockton, Linda S. Cook, Anthony M. Magliocco, Carrie S. Shemanko, Hans J. Vogel, Momtafin Khan, Karen A. Kopciuk

**Affiliations:** 1 American Institute for Cancer Research, Arlington, VA 22209, USA; n.brockton@aicr.org; 2 Department of Epidemiology, Colorado School of Public Health, University of Colorado, Aurora, CO 80045, USA; linda.cook@cuanschutz.edu; 3 Protean Biodiagnostics, Orlando, FL 32827, USA; magliocco@proteanbiodx.com; 4 College of Medicine, University of Central Florida, Orlando, FL 32827, USA; 5 Department of Biological Sciences, Faculty of Science, University of Calgary, Calgary, AB T2N 1N4, Canada; shemanko@ucalgary.ca; 6 Arnie Charbonneau Cancer Institute, University of Calgary, Calgary, AB T2N 4N1, Canada; vogel@ucalgary.ca; 7 Biochemistry Research Group, Department of Biological Sciences, Faculty of Science, University of Calgary, Calgary, AB T2N 1N4, Canada; 8 Department of Biochemistry and Molecular Biology, Cumming School of Medicine, University of Calgary, Calgary, AB T2N 4N1, Canada; 9 Department of Cancer Epidemiology and Prevention Research, Cancer Care Alberta, Alberta Health Services, Arthur JE Child Comprehensive Cancer Centre, 7th Floor, Room YC071 414, 3395 Hospital Drive N.W., Calgary, AB T2N 5G2, Canada; 10 Department of Community Health Sciences, University of Calgary, Calgary, AB T2N 4Z6, Canada; 11 Department of Oncology, University of Calgary, Calgary, AB T2N 4N2, Canada; 12 Department of Mathematics and Statistics, University of Calgary, Calgary, AB T2N 1N4, Canada

**Keywords:** breast cancer, cohort, bone metastases, progastrin (hPG_80_), vitamin D, survival, treatment

## Abstract

Women diagnosed with early-stage breast cancer can develop metastatic disease following successful initial treatment, with bone being the most common site of metastases. The Breast to Bone (B2B) cohort study of early-stage breast cancer patients was established as a research platform to study the basic biology of breast cancer to bone metastasis and to identify the factors that could improve prevention, early detection and treatment for this debilitating and incurable disease. The B2B cohort includes 478 women diagnosed with incident primary breast cancer (stages I to III) who were recruited from Calgary, Alberta and surrounding areas between February 2010 and July 2015. Four projects have been conducted to date, utilizing data and samples from this cohort. These studies have found the following: (a) women with insufficient or deficient levels of vitamin D (25[OH]D) concentrations in pretreatment serum samples had larger tumors and higher breast cancer grades, (b) several metabolomic biomarkers and cytokines were associated with tumor characteristics and time to recurrence, (c) additional biomarkers were found to be predictive for the high risk of bone metastasis and (d) circulating progastrin (hPG80) was associated with multiple survival outcomes. These research studies and future ones will provide new evidence on bone metastasis etiology in women diagnosed with early-stage breast cancer, improve identification of those at high risk and contribute to personalized treatment and prevention options.

## 1. Introduction

Breast cancer is the most common cancer afflicting Canadian women [1,2]. In 2024, an estimated 30,500 Canadian women will be diagnosed with breast cancer, and 5500 will die from it [1,2]. In a recent Alberta-based study, the incidence of late-stage (stages III and IV) breast cancers between 2006 and 2017 decreased at an annual rate of 0.8% (*p* = 0.3), and breast cancer-related deaths decreased at an annual rate of 2.3% (*p* < 0.001), respectively [3]. However, even with early detection from organized breast cancer screening programs and improved treatments, metastatic disease continues to cause substantial morbidity and mortality months or decades after the initial diagnosis [4,5].

Early studies estimated that 20 to 30% of women diagnosed with breast cancer will develop metastatic disease after their treatment [6,7], with 70% of metastases found in bone [8,9]. The median survival was approximately two years [10,11]. Recent studies of breast cancer patients diagnosed with stages I–III found that approximately 14% will develop bone metastasis by 15 years of follow-up [12], while a systematic review estimated that 12% of patients would develop bone metastases during a median follow-up of 60 months [13]. The predominant mechanistic hypothesis for breast to bone metastasis is the “seed and soil” hypothesis, which posits that bone is a favorable microenvironment for breast tumor cells to survive and proliferate, similar to how a seed will grow in fertile soil [14,15]. On the other hand, the mechanical mechanism hypothesis proposes that the pattern of blood flow determines metastasis sites [16]. Both of these hypotheses may work in complementary roles to disseminate tumor cells [14,16]. Bone-derived chemokines may also play a role in attracting breast cancer cells to the bone [17]. Bone metastasis has also been identified as a launch site for the development of other metastatic lesions to organs other than bone, shortening survival [18,19,20].

Although survival durations have improved, for patients with distant breast cancer recurrence, 20 to 30% of women with early breast cancer still die because of metastatic breast cancer [4,7,21]. Currently, no reliable prognostic factors exist that could target surveillance to those most likely to experience distant metastases. Micrometastases have been detected in the bones of 12 to 45% of breast cancer patients at diagnosis [22], but their presence is not strongly associated with the risk of distant relapse. Furthermore, diagnoses of micrometastases require a painful bone marrow aspiration procedure, which may additionally cause anxiety and distress [23].

Risk factors increasing the risk for the development of bone metastasis include menopausal status, larger tumor size and a greater number of cancerous axillary lymph nodes [24]. A meta-analysis found that PR-positive breast cancers have a lower risk of bone metastasis, while HER2-positive, lymph node metastasis-positive, nonlobular, or ductal breast cancers and increasing tumor stage have a higher risk of bone metastasis [25]. There is a continuing unmet need for prognostic biomarkers to determine who is at greater risk of bone metastasis and provide women with breast cancer with better and personalized tertiary preventive treatments.

The primary goals of the Breast to Bone (B2B) research program are to investigate the basic biology of breast cancer to bone metastasis and to identify lifestyle, pathological, clinical and therapeutic factors, aiding in the prevention, early detection and successful treatment of debilitating osseous lesions. The purpose of this paper is to provide the baseline characteristics of the B2B cohort and highlight some of the research studies conducted with these data.

## 2. Materials and Methods

### B2B Cohort Creation

The B2B research program developed and implemented a population-based rapid ascertainment of biopsy-confirmed breast cancer to establish a prospective cohort of women with incident breast cancer. Between February 2010 and July 2015, 478 women diagnosed with an incident of primary breast cancer were enrolled from Calgary, Alberta and surrounding areas out of 775 eligible women. The eligibility criteria included an incident American Joint Committee on Cancer (AJCC 7th edition) stage I-IIIC breast cancer diagnosis, being assigned female at birth, being between the ages of 18 and 80 years old at diagnosis, and with no prior cancer diagnosis. Patients were not excluded on the basis of comorbidities, but comorbid conditions were recorded at baseline.

All invited participants had to provide a pre-surgical blood sample at the time of diagnosis at any Calgary Laboratory Services location [26]. The baseline collection was highly standardized and consisted of a 60 mL blood sample collected in six 6 mL Red Top (clot activator) vacutainers and four 6 mL Lavender Top (EDTA) vacutainers. Within 24 h, the vacutainers were refrigerated and transported to a central processing laboratory for fractionation by centrifuge to yield a total of 48 aliquots comprised of 26 serum, 14 plasma, 4 buffy coats and 4 red blood cells (400–500 μL per aliquot) in 1 mL of Matrix^®^ 2D barcoded tubes (Thermo Fisher Scientific Inc., Rochester, NY, USA). The interval from venipuncture to processing was recorded for every sample. The aliquots were immediately transferred to −80 °C freezers for storage until use. At the time of blood collection, the participants also completed a short blood questionnaire to record information regarding their fasting status, recent smoking, medication and supplement use, family history of cancer and menstrual status [26].

**Figure 1:**
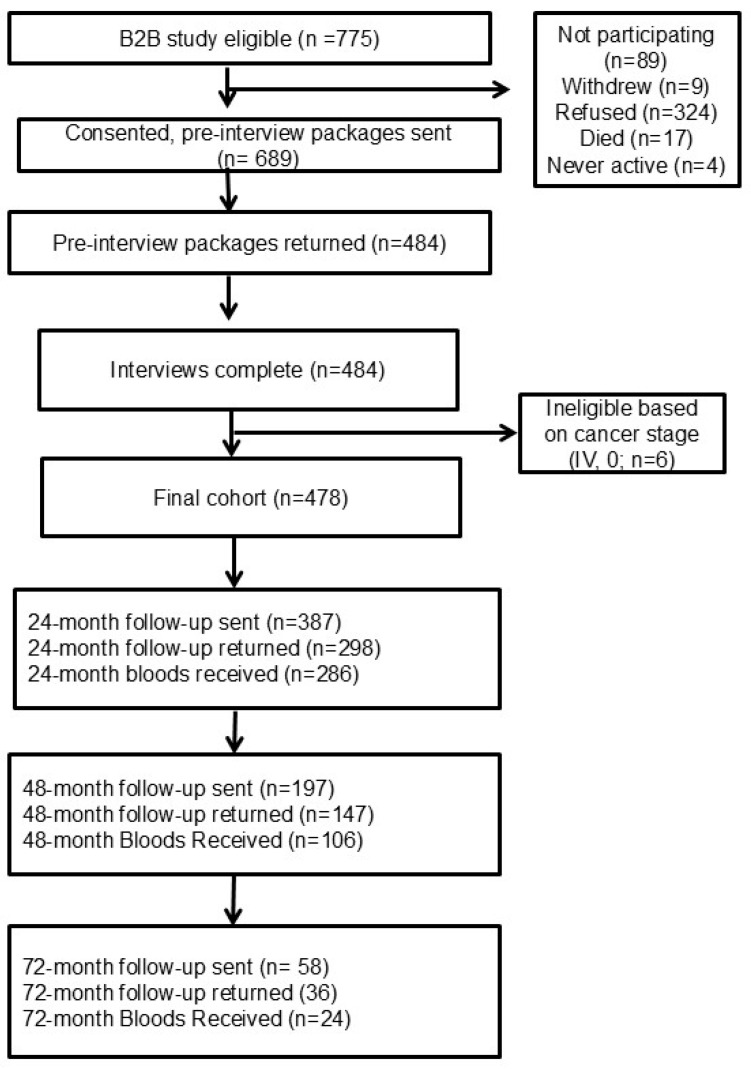
B2B recruitment and follow-up flowchart, Alberta, Canada, 2010–2024.

At baseline, an interviewer collected the health and lifestyle questionnaire data by utilizing the following instruments: Canadian Dietary History Questionnaires (CDHQ) (I/II) [27,28], Past Year Total Physical Activity Questionnaire (PYTPAQ) [29], an abbreviated Sun Exposure Questionnaire [30] and the B2B Baseline Interview [26]. The B2B Baseline Interview collected information regarding pregnancy and menstruation, menopausal status, hormone replacement therapy, birth control and hormone contraceptive use, personal health history/comorbidities, medications (over-the-counter and prescription), vitamins, minerals and herbal supplements, mobility and physical activity, sun exposure, diet history, family history of cancer, smoking habits, alcohol consumption history and demographic information. Follow-ups were done at 24, 48 and 72 months post-diagnosis, when the CDHQ I/II, and PYTPAQ questionnaires we sent to the participants to complete as well as the blood samples were obtained. Figure 1 presents the recruitment information at baseline and during the 24-, 48- and 72-month follow-ups. In total, 478 pre-surgical blood samples were collected at baseline, while at the 24-, 48- and 72-month follow-ups, 234, 106 and 24 blood samples, respectively, were collected (Figure 1). All the women had breast cancer surgery following their diagnosis, with 235 women also receiving initial chemotherapy and 315 receiving initial radiation therapy following their diagnosis. Further information regarding the cohort creation and initial projects can be found in the cohort establishment paper [26].

## 3. Results

### 3.1. B2B Cohort Description

The median age of the B2B cohort participants was 56 years of age, ranging from 24 to 80 years of age. Most of the 478 participants had at least some college or university education (*n* = 304, 63.6%), were married or in a common law union (*n* = 326, 68.2%), were from a European background (*n* = 261/289 responses, 90.3%) and had an annual household income between 50,000 and 100,000 CAD (*n* = 146, 30.5%) (Table 1).

Most of the participants were diagnosed as AJCC 7 stage I (*n* = 247, 51.7%) and stage II (*n =* 194, 40.6%) and had no lymph nodes detected (*n =* 328, 68.6% Table 2). The majority were classified as luminal A subtype (*n =* 345, 72.2%), with the remaining women classified as having luminal B (*n =* 65, 13.6%), triple-negative (*n =* 30, 6.3%) and HER-2 (*n =* 18, 3.8%) subtypes. There were similar numbers of women classified as medium and high grade on the Bloom–Richardson scale, *n =* 197 (41.2%) and 194 (40.6%), respectively.

We examined the past health history and lifestyle factors of the B2B cohort at baseline and presented some selected characteristics in Table 3. Just over one-third of the B2B study participants were either smokers (*n* = 34, 7.1%) or ex-smokers (*n =* 147, 30.8%). However, 57.7% (*n =* 276) of women did not report their smoking status. Most participants (*n =* 384, 80.3%) reported consuming at least six alcoholic drinks in any given year. About one-fifth reported a family history of breast cancer (*n =* 95, 19.9%). Two-thirds were post-menopausal (*n =* 329, 68.9%), and the most frequent number of pregnancies was two (*n =* 143, 29.9%), with 65 reporting being nulliparous (13.6%). Few women had a hysterectomy (*n =* 95, 19.9%) or bilateral oophorectomy (*n =* 29, 6.1%), but most did use birth control (*n =* 375, 78.5%). For hours spent sitting at work, 27.8% (*n =* 133) reported sitting 2.1–6 h per day, while 32.6% (*n =* 156) reported not sitting at all at work. About half of the women slept 5 to 7 h each night (*n =* 222, 46.4%), while about a third of the (*n* = 147, 30.8%) women reported using a tanning salon or sun lamp in their lifetime. About one-third of the women were normal weight (*n =* 158, 33.1%) or overweight (*n =* 144, 30.1%), and nearly half (*n =* 236, 49.8%) of the cohort also had a high waist-to-hip ratio (>0.85 cm).

**Figure 2:**
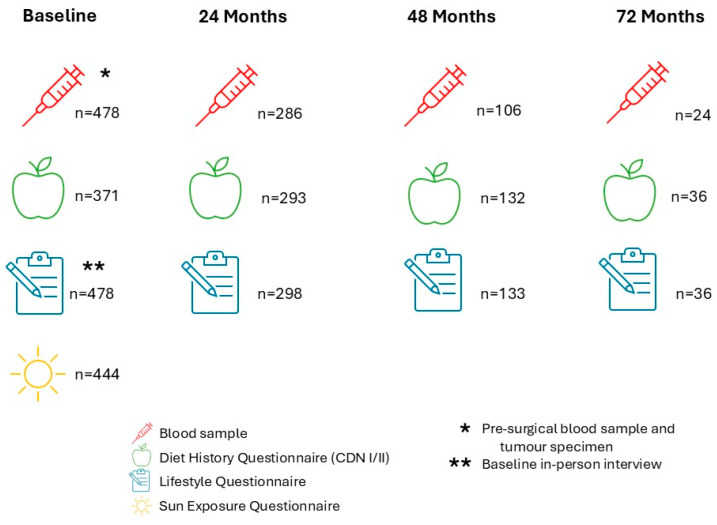
Infographic of data and samples collected in the B2B cohort at baseline and 24-, 48- and 72-month follow-ups.

Figure 2 provides details on the blood samples and information available at each time point in the study. Lifestyle questionnaires were administered in person at baseline and were self-reported at subsequent follow-up time points.

### 3.2. Projects Utilizing Baseline Data and Biological Samples

Several research projects have been conducted using the data and biological samples collected at baseline from the B2B cohort. Although some of these projects were proposed in the creation of the B2B cohort [26], some arose in subsequent collaborations.

The association of vitamin D with tumor characteristics in pre- and post-menopausal women was recently investigated [31]. Women with insufficient or deficient levels of vitamin D, especially pre-menopausal women, had tumors that were larger in size and a higher grade. Vitamin D has an anticarcinogenic effect that operates through multiple mechanisms, including pro-differentiation and anti-proliferation [32]. In addition, vitamin D has been implicated in several chronic diseases that have an inflammatory component, such as rheumatoid arthritis and cancer [33]. Thus, a suspected etiology of bone metastases involves an inflammatory component that is compatible with observed immunosuppressive and anti-inflammatory activities of the active metabolite of vitamin D [34]. The role of vitamin D in breast cancer etiology and metastases to bone continues to be complex and not well understood. However, analysis of tumor characteristics and baseline serum vitamin D levels in the B2B cohort revealed that a lower vitamin D status was associated with larger tumor sizes and higher grades [31]. Although more research is needed, vitamin D supplementation is a potentially cost-effective prevention strategy that could easily be implemented.

Nuclear magnetic resonance (NMR) spectroscopy and metabolic markers for the bone metastasis project utilized NMR- metabolomics [35] and human biofluids (serum) to generate metabolite profiles using the B2B baseline samples. Metabolomic biomarkers have been used for disease diagnosis and prognosis, reducing the invasiveness, costs and complexity of tissue sample collection. Metabolomic biomarkers were assessed for their association with tumor characteristics in the B2B cohort. Their association, as well as 17 cytokines, are currently being evaluated as prognostic factors for the development of metastases, with a manuscript in preparation. Significant prognostic biomarkers for recurrence can provide additional biological insights into the development of bone and other sites of metastases, with the potential for early detection of women at risk, as well.

Despite being incurable, up to 70% of bone (only) metastasis patients are alive after 10 years [18]. Quality of life is, therefore, an important consideration, given the severe morbidity of bone metastasis, though currently, there is no molecular test available for which to test patients and change their treatment options. Based upon the earlier discovery where it was identified that high levels of the hormone prolactin receptor on breast cancer cells of the primary tumor were associated with a shorter time to bone metastasis [36], additional research [37] resulted in the identification of biomarkers predictive of a high risk of bone metastasis. The available clinical information, tumor material and blood products in the B2B cohort provide a rich source of patient information and molecular data for biomarker generation and validation.

A fourth project evaluated whether circulating progastrin (hPG_80_) was prognostic for women with breast cancer in the B2B cohortThis biomarker has been detected at significantly higher concentrations in cancer patients than in healthy blood donors [38,39], and hPG_80_ has also been associated with survival outcomes in people diagnosed with renal and hepatocellular cancers [40,41]. This project found a positive association between hPG_80_ levels and an increased risk of overall survival, time to recurrence (any location) and disease-free survival outcomes in women in the B2B cohort [42]. This finding may elucidate a new prognostic biomarker for clinicians to utilize in treatment selection and follow-up care of breast cancer patients.

## 4. Discussion

The B2B cohort is an Alberta-based prospective study, with 478 female participants with breast cancer, that collected information on demographics, health history, dietary history, physical activity and sun exposure at baseline. The cohort participants had mostly early-stage breast cancer, were diagnosed at a median age of 56 years and were generally well-educated with modest incomes. Most were classified as normal or overweight based on their BMI, were post-menopausal and were without a family history of breast cancer. Additional follow-up data and biological samples were obtained from some of the women in the cohort at 24, 48 and 72 months post-diagnosis. As of December 2023, 64 participants have died, with 37 deaths due to breast cancer. Four projects have been completed using the baseline data and biological samples: elucidating information on the relationship of tumor characteristics with vitamin D status; NMR metabolomics and time to recurrences; biomarkers discovered subsequent to the characterization of the hormone prolactin receptor; and hPG80 and survival outcomes. These, as well as ongoing and future projects, could identify women at high risk of recurrence, especially for bone metastases, that could lead to more personalized treatments and prevention.

Several international breast cancer cohorts have been created, including the French Early Breast Cancer Cohort (FRESH), Life After Cancer Epidemiology (LACE), the Health, Eating, Activity, and Lifestyle (HEAL) and the Pathways studies. FRESH was created to research the adverse effects, morbidity, mortality, monitoring of care consumption and/or medical economics in a large (nearly 235 386) cohort of French breast cancer patients [43]. The information collected in the FRESH study only included sociodemographic variables, breast cancer subtypes and nodal status [43] and did not specifically assess metastases. The LACE study, based in the United States, was established to examine the quality of life and long-term survival of breast cancer patients in relation to modifiable risk factors [44]. It collected information on demographics, medical history, anthropometry, diet, supplements, physical activity and quality of life [44], but not any biological samples. Similarly, the HEAL study was focused on studying levels of physical activity before and after a breast cancer diagnosis because previous literature suggested that increased body weight may be a risk factor for breast cancer recurrence and reduced survival [45]. The HEAL study included 812 patients with incident breast cancer and determined that physical activity levels were reduced significantly after diagnosis, implying a potential for greater weight gain [45]. Finally, the Pathways study is a California-based prospective cohort study with over 1500 breast cancer participants [46]. The Pathways study examines the impact of lifestyle factors on prognosis, and while the study does collect blood samples at baseline, some may be collected before treatment, while some may not [46]. While all these international studies provide valuable information pertaining to breast cancer, they do not collect information on breast to bone metastasis—this is the focus of the B2B cohort. Additionally, three [43,44,45] of the four international studies described above did not collect the blood samples that were collected in the B2B cohort. The results from these international studies may not be generalizable to the Canadian population.

The B2B cohort has important strengths compared to other prospective Canadian cohorts, such as Alberta’s Tomorrow Project (ATP), which is also part of the Canadian Partnership for Tomorrow’s Health Project (CanPath). For example, ATP collected biological samples but has not run similar assays for cytokines, metabolites and common lab measures thus far for all participants. Furthermore, while serum vitamin D levels are available in the B2B cohort, only self-reported vitamin D consumption is available in the ATP and CanPath data, which is prone to measurement errors. ATP has collected blood samples from participants, some before their breast cancer diagnosis and some after, but it is unknown if these samples were pre-surgery or treatment or not [47]. This contrasts with the collection of pre-surgical blood samples collected at diagnosis in the B2B cohort. Pre-surgical blood samples have many advantages: they are collected when the tumor is still in place, so potentially relevant biomarkers are intact, and blood samples are unaltered by the inflammatory response to surgical removal of the tumor and subsequent wound healing responses. Regardless, all these cohorts assess exposures before outcomes, so there is clarity in the temporal sequence of exposures and outcomes.

A limitation of the B2B cohort, however, is the incomplete follow-up, which resulted in fewer participants contributing data and samples after enrolling in the B2B cohort. In this study, 478 participants were recruited; however, by the 24-month follow-up, only 295 participants provided updates to their health, lifestyle and diet histories. A total of 234 of these participants also provided another blood sample. Even fewer women were contacted at 48 and 72 months, in large part because of a lack of funding. Another limitation is the missing information on some health and lifestyle characteristics that affect the factors that can be included in analyses or studied. As a predominately Caucasian cohort, the B2B cohort does not reflect the racial diversity of the population of women diagnosed with breast cancer diagnosed in Alberta and elsewhere. The homogeneity of this cohort could limit the generalizability of findings from the data and samples to other population groups.

Another Alberta-based prospective breast cancer cohort is the Alberta Moving Beyond Breast Cancer (AMBER) study, which recruited 1528 newly diagnosed cases between 2012 and 2019. [48]. The AMBER study focuses on physical activity, sedentary behavior and health-related fitness on survival outcomes. The same recruitment process was used for both studies, as described in the cohort development paper [26]. However, the AMBER study was not able to collect pre-surgical blood samples from all participants, unlike the B2B project, because that cohort was conducted in Edmonton, as well, where pre-surgical blood samples could not be collected.

A recent study examined the role of race/ethnicity on breast cancer characteristics by linking several Canadian databases, including the Health and Environment Cohorts (2006 and 2011), Cancer Registry and Vital Statistics [49]. The characteristics in this Canadian cohort were similar to the participants in the B2B study, although the percentage of stage I diagnoses is slightly lower (46.5% versus 51.7% in the B2B study). The percentage of luminal A subtypes in white women (62.3%) was also slightly lower than in the B2B study (72.2%), which is predominately white. The median age of 63 years for white Canadian women diagnosed with breast cancer was slightly older than the median of 56 years of age for the B2B participants, but that could be due to the younger population in Alberta [50]. This Canadian study did find that non-white women were diagnosed at younger ages and had lower incidence and mortality rates than white women. A limitation of the B2B cohort is the inability to assess the role of ethnicity on the development of metastases, as the cohort is largely Caucasian due to the population composition for this time period in Alberta.

Some studies suggest that there are differences in the incidence of breast cancer in ethnicities attributable to biological, social or healthcare system factors. [51,52,53,54]. Another recent Canadian study suggested that disparities exist in the diagnostic process between immigrant women of different racial backgrounds compared with long-term residents of Ontario and British Columbia [55]. A systematic review examining the rate of recruitment into research studies for racial minorities (African-American, Asian-American, Latino and Pacific Islanders) revealed several barriers to participation [56]. Barriers included a perception that the research will benefit Caucasian individuals rather than racial minorities, concerns about participation costs and time conflicts, lack of access to information about research and research methods, and finally, stigma about the diseases studied [56]. Increasing the recruitment rates of racial minorities would improve the generalizability of the results from cohorts such as the B2B. Despite these limitations, the B2B’s strengths and extensive data on many social determinants of health and etiological risk factors provide researchers with a platform to build on existing knowledge and determine new relationships between the variables of interest and breast cancer outcomes such as metastasis and/or recurrence.

## 5. Conclusions

The focus of the B2B cohort is on identifying the biological mechanisms for the development of metastases and identifying breast cancer cases at diagnosis who may be at risk of local, regional or distant recurrence, particularly to bone. The B2B cohort is building on its premise to improve our understanding of the biology of bone metastasis in breast cancer patients and to identify risk factors to prevent, detect and treat bone metastases in the future. In addition to this main focus, the B2B cohort has been utilized for other purposes, such as evaluating biomarkers associated with tumor characteristics as well as survival outcomes. The B2B cohort offers the opportunity to test emerging hypotheses in a timely manner. Validating these findings could provide a new basis for individualized treatments in conjunction with prognostic and diagnostic methods developed through the projects in the B2B cohort. The need to identify women diagnosed with early-stage breast cancer who are at high risk of developing metastatic disease will provide more opportunities for personalized treatment and prevention options.

## Figures and Tables

**Table 1:** Demographic characteristics of the B2B cohort at baseline (N = 478), Alberta, Canada, 2010–2015.

Characteristic	N	Percentage (%)
Age at diagnosis (years)		
<40	29	6.1
40–49	86	18.0
50–59	145	30.3
60–69	117	24.5
>70	91	19.0
Missing	10	2.1
Education		
Less than high school	19	4.0
High school	79	16.5
Vocational or technical	43	9.0
Some college or university	75	15.7
University undergraduate degree or college diploma	188	39.3
University graduate degree	41	8.6
Missing	33	6.9
Marital Status		
Married/Common Law	326	68.2
Widowed	27	5.6
Divorced/Separated	56	11.7
Other/Missing	69	14.4
Ethnicity		
European	261	54.6
Aboriginal	5	1.0
Asian	16	3.3
South American	6	1.3
African	1	0.2
Missing	189	39.5
Income (CAD)		
<50,000	58	12.1
50,000–100,000	146	30.5
100,000–200,000	126	26.4
>200,000	69	14.4
Missing	79	16.5

**Table 2:** Tumor characteristics of B2B study participants, Alberta, Canada, 2010–2015.

Characteristic	N	Percentage (%)
AJCC * 7 stage		
I	247	51.7
II	194	40.6
III	37	7.7
Bloom–Richardson (BR) grade		
Low	70	14.6
Medium	197	41.2
High	194	40.6
Unknown	5	1.0
Missing	12	2.5
Lymph nodes		
N0	328	68.6
N1mi	115	24.1
N1	19	4.0
N2	1	0.2
N3a	2	0.4
N3c	1	0.2
Missing	12	2.5
HER2 receptor		
Positive	84	17.6
Negative	372	77.8
Indeterminate	4	0.8
Not done	5	1.0
Missing	13	2.7
Estrogen receptor		
Positive	408	85.4
Negative	56	11.7
Not done	2	0.4
Missing	12	2.5
Progesterone receptor		
Positive	377	78.9
Negative	86	18.0
Not done	3	0.6
Missing	12	2.5

* AJCC = American Joint Committee on Cancer.

**Table 3:** Selected health history and lifestyle characteristics of the B2B cohort at baseline (N = 478).

Characteristic	N	Percentage (%)
Smoking Status		
Occasional smoker	7	1.5
Ex-occasional smoker	14	2.9
Current smoker	34	7.1
Ex-smoker	147	30.8
Missing	276	57.7
Ever had 6 drinks of beer, wine or liquor in any given year?		
No	60	12.6
Yes	384	80.3
Missing	34	7.1
Family history of breast cancer		
No	349	73.0
Yes	95	19.9
Missing	34	7.1
Number of pregnancies		
0	65	13.6
1	51	10.7
2	143	29.9
3	102	21.3
4	48	10.0
>4	36	7.5
Missing	33	6.9
Menopausal status		
pre-menopausal	116	24.3
post-menopausal	329	68.9
missing	33	6.9
Both ovaries removed		
No	416	87.0
Yes	29	6.1
Missing	33	6.9
Hysterectomy		
No	217	45.4
Yes	95	19.9
Missing	166	34.7
Birth control used ^a^		
No	70	14.6
Yes	375	78.5
Missing	33	6.9
Hours sitting at work ^b^		
0	156	32.6
0.1–2	77	16.1
2.1–6	133	27.8
>6	78	16.3
Missing	34	7.1
Hours sleeping ^c^		
<5	50	10.5
5–7	222	46.4
>7	140	29.3
Missing	66	13.8
Ever used a sun lamp, or gone to a tanning salon or solarium		
No	297	62.13
Yes	147	30.75
Missing	34	7.11
BMI category		
Underweight (BMI < 18.5)	10	2.1
Normal (18.5 ≤ BMI < 25)	158	33.1
Overweight (25 ≤ BMI < 30)	144	30.1
Obese class I (30 ≤ BMI < 35)	67	14.0
Obese class I (30 ≤ BMI < 35)	30	6.3
Obese class III (BMI ≥ 40)	23	4.8
Missing	46	9.6
Waist-to-hip ratio category (cm)		
<0.8	95	19.9
0.8 to 0.85	100	20.9
>0.85	236	49.4
Missing	47	9.8

^a^ Birth control used includes any method of birth control in the form of hormone contraceptives, pills, shots or implants; ^b^ On average, in the two years prior to your breast cancer diagnosis date, how many hours in a day did you spend in the following sitting activities?; ^c^ On average, in the two years prior to your breast cancer diagnosis date, how many hours in a day (over a 24 h period) did you sleep (including naps)?

## Data Availability

The data presented in this study can be available upon request from the corresponding author if collaborations are established with the study lead investigator due to the legal requirements based on the consent of participants.
